# TIAP: an observational procedure for assessing family relationships: a clinical case from the parenting evaluation context

**DOI:** 10.3389/fpsyg.2024.1330115

**Published:** 2024-05-17

**Authors:** Ada Cigala, Elena Venturelli, Laura Fruggeri

**Affiliations:** ^1^Department of Humanities, Social Sciences and Cultural Industries, University of Parma, Parma, Italy; ^2^Bologna Family Therapy Center, Bologna, Italy

**Keywords:** family relationships, parenting evaluation, observational methods, family assessment, family functioning, triadic interactions

## Abstract

TIAP is an observational procedure to assess family functioning detecting simultaneously the role of each participant and the interdependence of relational behaviors. In particular, the procedure requires family members to play according to different interactive configurations (parent1-children; parent2-children, all together, children and parents as separate units) and therefore different microtransitions from one configuration to another. As such, the procedure allows to study how family members coordinate to maintain stability, promote change, and encourage members to explore different interactive configurations within the family system. TIAP has been validated through several studies conducted with different non-clinical groups of families that have highlighted the salient aspects of family functioning, and significant correlations with variables external to the family system, such as children’s social–emotional competence in the educational context. This paper focuses on the use of TIAP in the contexts of assessing parental competence. Specifically, the article aims to describe, through the reference to a clinical case, the results emerged from a study conducted with 33 families involved in a parenting assessment process. The study is part of a broader collaborative project between the Child and Adolescent Neuropsychiatry Clinic of the Italian National Health Service in Parma, the University of Parma, and the Bologna Family Therapy Center. TIAP was administered to all the families involved as a complement to other tools routinely used for all cases handled by the professionals of the clinic. The coding system includes different indices. Some analyze the interactive family modes: family coordination (mutual attention and responsiveness), the responses to potentials for change (disregard, absorption, amplification), and intra-familiar exploration. Other indices concern the quality of the interactions: the relational triadic dynamic of microtransition (detaching-entrusting-welcoming-joining) and the consistency/inconsistency of the communication channels. The results highlighted how TIAP makes it possible to identify the specific interactive modalities of the different members and their interdependence and reciprocity, favoring the identification of both family weaknesses and family resources, including the children’s contribution. Furthermore, the general data trend showed that TIAP indices detect some important prognostic elements capable of guiding the court’s decisions.

## 1 Introduction

In the field of family studies, various analogical methods using narrative, symbolic, metaphorical, and observational tools have been elaborated to detect the representations that family members have of themselves as a group, to explore the dynamics between members, and, in psychotherapy, to introduce elements that can foster change (for a review see Kerig and Lindahl, 2001; Di Nuovo, 2015; Venturelli et al., 2016, 2022). The explicit need is to focus on research, evaluation, and intervention procedures consistent with the relational, systemic, and processual nature of the object of analysis (Lanz and Rosnati, 2002; O’Brien, 2005; Lanz et al., 2015).

Among the several tools, observational methods are particularly adequate to analyze family interactions, dynamics, and processes. Indeed, they allow to directly observe how one’s behavior interrelates with others’; to study how the different interactive behavioral sequences unfold across time, thus, they allow to observe and describe the ongoing family processes (Margolin et al., 1998). The observational methods respond to the need underlined by many scholars and clinicians to use tools able to acknowledge the complexity, processes, and interdependence of family relationships (Fivaz-Depeursinge and Corboz-Warnery, 1999; McGoldrick et al., 2011; McHale and Lindahl, 2011; Venturelli et al., 2016; Walsh, 2016; Venturelli et al., 2022).

TIAP (*Triadic Interactional Analytical Procedure*) (Venturelli et al., 2022) is an observational instrument for assessing family functioning. It is the result of a research composed of several studies conducted with non-clinical families and through various experiments in different applicative contexts. It is a research process that has taken place thanks to the convergence of different perspectives (social, developmental, and clinical), and that has focused on different yet connected constructs such as configurations, microtransitions, family coordination, potential spaces for change, stability, change, intrafamilial exploration that have proved particularly useful in analyzing families’ functioning in a daily and process-oriented perspective (Cigala et al., 2014, 2018).

### 1.1 Theoretical premises of TIAP

The theoretical framework of the research that led to the development/elaboration of TIAP is based on the following points:

#### 1.1.1 The triad as a minimum unit of analysis

The triadic approach provides a method of study that simultaneously considers the position of individuals in the system, the interpersonal relationship each one has with another, the relational dynamics between all and the circularity between these different levels (individual, dyadic, systemic). Triadic models are consistent with a systemic approach -traditionally and fruitfully used for the study of family dynamics- and deepen it because they make it possible to analyze interpersonal interactions, focusing on the active role of all members, without losing sight of the whole group (Parke, 1988).

Through the observation of a triad, it is possible to detect the behavior of people who find themselves from time to time in the position of those who are directly involved with another, while the third observes; in the position of those who observe the other two engaged in a reciprocal exchange, and therefore peripheral to that exchange; finally, in the position of those who interact simultaneously with all the others. The analysis of triadic situations makes it possible to detect important psycho-social abilities of the participants such as: the ability to stay in the relationship with another, the ability to stay out of it, and the ability to interact with two partners at the same time without shirking or excluding anyone. These capacities emerge as interconnected in the triadic dynamic and constitute the outcome of a coordination between all the components of the triad. In fact, on the one hand, the capacity to be in the relationship is an individual capacity that can be expressed through behaviors such as paying attention, responding to the interlocutor’s needs, emotionally connecting with the partner, leaving the third party in a peripheral position, i.e., avoiding soliciting him/her to participate in the ongoing dyadic exchange. On the other hand, the capacity to be in a relationship may be favored or hindered by the position assumed by the peripheral third party, who, reciprocally, may tolerate remaining on the margins or instead intervene or self-exclude. But the peripheral position of one of the interlocutors will be more easily maintained the more the interaction of the others is perceived as harmonious. Moreover, the ability to interact with more than one interlocutor implies that each one avoids capturing one of the interlocutors within a dyadic exchange, excluding the third; but this is also facilitated by the condition that no one, by withdrawing from the interaction, ends up authorizing others to engage in an exclusive dyadic exchange (Fruggeri, 2002).

In triadic contexts, it is also possible to experience distancing within a safe context so that detachment does not produce traumatic experiences but becomes an opportunity to stimulate growth and the expansion of relational opportunities. As well as there is the possibility of distancing oneself from a network of relationships without experiencing the discomfort of abandonment. The triad, unlike the dyad, constitutes a context in which the detachment from someone can be contingent on reliance on someone else, thus filling that void that may occur while passing from one involvement to another. The triadic context allows for a relational coordination in which the one who separates can entrust his or her interlocutor to a third party who is in turn ready to welcome the one who has been left (Fruggeri, 2002; Cigala et al., 2013, 2014). In a dyadic context, separation can take on the connotation of abandonment; in a triangular/systemic context, detachment is the complementary process of entrusting to others and thus the precursor of new relational involvements.

In the analysis of triadic forms of interactions, it is possible to focus on the interdependence of relational contexts that characterize families. The meaning assumed by a relationship between two components depends both on the interaction in which they are directly involved, and on the quality of the relationships they experience with other components; in turn, what is negotiated in terms of the quality of the relationship between two interlocutors will have a repercussion on the other relationships in which they are directly or indirectly involved. In triadic relational contexts, processes are co-evolutionary in that, due to the interdependence that defines them, a change that occurs in one dyadic relational context will have repercussions in all the other relational contexts in which the members of the dyad are involved (Fruggeri, 2018; Venturelli, 2018; Fruggeri et al., 2023).

#### 1.1.2 Families in everyday life

The study of family processes has gained benefits from the approach based on the analysis of everyday practices (Fiese, 2006; Emiliani, 2013). It is a research perspective that focuses on how family members coordinate in dealing with their tasks. The focus is thus on “the how” of family life instead of “the what”; the attention is then paid to processes, interactive dynamics, and relational patterns (Fiese, 2006; Emiliani, 2008).

In families, everyday practices are characterized by different forms of triadic interactions, and by microtransitions that mark the passage from one form of triadic interaction to another. These microtransitions involve deconstructions, reconstructions, further deconstructions and reconstructions of interactive configurations (Cigala et al., 2009). For example, consider a family scenario in which mother and child are playing together while the other mother is in the same room sitting on the couch reading a book; at a certain point she turns to her partner and asks her to sit next to her, because she wants to show her a sentence. The first mother stops playing with the child, joins the partner and starts talking to her. The child continues to play. In this moment of family life, it is possible to identify the microtransition from an interactive configuration in which the second mother has a peripheral position to a new configuration in which the little girl assumes a peripheral position. It is conceivable that shortly thereafter, the child stops playing, joins the mothers who interrupt what they are doing to involve themselves with their daughter, and then later they all move on to yet another configuration in which the first mother goes to set the table for dinner and the second mother accompanies the child to wash her hands.

But think also of the deconstructions and reconstructions of interactional configurations involved in the day-to-day care of a child by the kindergarten teachers or grandparents. Microtransitions from one interactional configuration to another are crucial moments that require the ability of the members to coordinate with each other; they involve complex capacities such as those of separating and rejoining with another partner, of tolerating being peripheral with respect to an interactional scene, as well as of tolerating the other being in a peripheral position, of paying attention to the signals of others, etc. The alternation and succession of many micro-transitions marks the unfolding of daily family life through which identity, relationships, personal, interpersonal, and social skills are built.

#### 1.1.3 The processes that define the quality of family functioning

From research conducted with non-clinical families, certain processes were found to be particularly significant in discriminating different styles of family functioning. They are family coordination, family stability, family change, intra-family exploration (Venturelli et al., 2022).

*Family Coordination* refers to an interactive form whereby a family member coordinates his or her own behavior (verbal, corporal, and expressive) with that of another family member who in turn interacts with a third (Westerman and Massoff, 2001). A triad is highly coordinated when all members are attentive to each other’s moves, notice them, realize that something has changed and organize themselves together in such a way as to arrive at a condition of new stability (new in the sense of another stability, which may be within the previous configuration or a different one). In other words, high triadic coordination allows each member to remain available to the information of the others and in connection with the others, so that the triad is ready to deconstruct and restructure the forms of interactions through which the everyday family life unfolds (Cigala et al., 2010).

*Family change* is meant as the relentless process that takes place in everyday family life, when members are constantly involved in situations that may require a re-organization of their relational and interactive patterns. Family microtransitions are those moments or micro-moments of everyday life when the members of a family negotiate, redefine, reorganize, readjust relational and behavioral roles, interactive modalities, reciprocal positioning, power relations, hierarchy and daily routines. In other words, microtransitions are local interactive moments or micro-moments through which family members construct what they are and what they are going to be (Breunlin, 1988; Cigala et al., 2013, 2014).

*Family stability* refers to how members coordinate their behaviors for the maintenance of the daily practices, routines and rituals that constitute the scaffolding of the development of the group and its components. The studies on family routines have shown how the maintenance of the family’s continuity/identity over time provides a secure context for its members, who can experience belonging and rely on clear rules and stable contexts of meaning (Fiese, 2006). Stability enables members to recognize a sense of belonging and the typical family interactional patterns. The constant search for stability and continuity is considered a protective factor of family well-being, as it increases the sense of security, belonging, cohesion, satisfaction (Fiese and Wamboldt, 2001; Emiliani, 2013) and strengthens the social skills of members, especially children (Spagnola and Fiese, 2007). Family stability is a state that needs to be continually constructed in front of the countless inputs coming from inside and outside the family. This is why we connote family stability as a process (Cigala et al., 2015, 2018).

*Intra-family exploration* (Byng-Hall, 1995a). The everyday manifold and ever-changing relational scenario described above implies that family members move constantly from one situation to another in a sort of a dance in which people connect and detach to join someone else, and eventually get all together. The exploration of all these different interactive configurations is a developmental task since family members experiment separations and joining, interact with more partners at the same time, take a central and a peripheral position, are involved in change processes and in the maintenance of stability; and in so doing they also develop the social abilities needed to explore the world outside the family.

TIAP has been specifically elaborated to operationalize these processes.

### 1.2 The triadic interactional analytical procedure

Based on the previous theoretical premises, TIAP is an observational procedure to assess family functioning detecting simultaneously the role of each participant and the interdependence of relational behaviors. TIAP analyses the interactive microanalytic processes that occur daily between family members, involving different verbal, gestural and expressive communicative channels.

TIAP has been validated through several studies conducted with different non-clinical groups of families that have highlighted the salient aspects of family functioning, and significant correlations with variables external to the family system, such as children’s social–emotional competence in the educational context (Cigala et al., 2013, 2014, 2018; Venturelli et al., 2016). Recently, TIAP has also been tested in the contexts of family therapy and parenting assessment with very interesting results (Venturelli et al., 2022).

#### 1.2.1 The TIAP task

The task requires family members to play according to different interactive configurations (one parent-children, the other parent-children, all together, children and parents as separate units). All family members are invited to sit around a table and are given the following instructions: “We are asking you to play together for approximately 20 min, in four different combinations: first a parent plays with the child whilst the other parent watches; next the other parent plays with the children while the parent previously involved watches; next, all of you play together; and finally parents talk with each other whilst the children play alone.”

Through the assigned task, taken in part from the Lausanne Trilogue Play procedure (Fivaz-Depeursinge and Corboz-Warnery, 1999), the family triads are asked to act in four different configurations and thus to deconstruct and re-construct their interactional configuration three times, accomplishing three transitions: from a configuration in which a parent plays with the children and the other parent watches [(P1-C) P2], to another in which the other parent plays with the children and the parent who had previously played is in a peripheral role [(P2-C)P1], to one in which they all play together [(P1-C-P2)], and finally to the configuration in which the parents interact whilst the children are in the peripheral position [(P1-P2)C].

This task allows the observation of a family while the members jointly reproduced, within a short time, different interactive situations that usually take place in everyday life. Each member of the family is asked to separate and join several times taking different interactive positions. Thus, this task allows to analyze how family members coordinate to maintain stability, promote change, and encourage members to explore different interactive configurations within the family system.

The play materials used must be suitable for the age of the children, unstructured and without specific purposes. It does not have to be a problem-solving task, since the aim is to observe how family members interact spontaneously, in the absence of specific goal. For example, Lego constructions fulfill both the age-appropriateness requirement for children and the absence of predefined objectives if they are without instructions (Venturelli et al., 2022).

#### 1.2.2 The TIAP coding system

Consistent with the previous premises, the daily interactions of a family can be conceptualized and represented as an interactive flow, characterized by some perturbations and by the responses to these perturbations.

**Figure 1:**
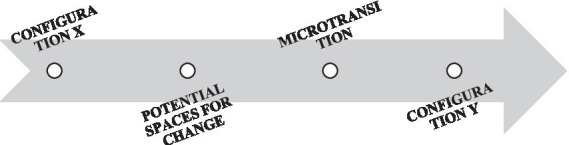
The interactive flow.

In the interactive flow that occurs daily between family members, which is reproduced in the TIAP task, it is possible to distinguish 3 different units of analysis: configurations, potential spaces for change and microtransitions (Figure 1).

Configuration refers to the interactive space in which individuals act jointly while maintaining the same interactive positions: active or peripheral. Potential for change refers to the verbal, corporal and expressive movements of any participant that, corresponding to a variation in her/his position, could bring about a change in the whole ongoing configuration. We called such movements potentials for change because the chances that they could trigger a variation in the configuration depend on the responses of the other partners in the interactive space. Three are the possible responses to the potentials for change: disregard when the potential for change falls in the void, it is not seen or it is voluntarily ignored; consequently, the ongoing configuration does not vary; absorption when one partner acknowledges the potential for change yet maintains her/his position in the ongoing configuration; amplification when the potential for change is noticed, fed back and amplified by a change in the position of everyone involved. In this case the potential for change becomes the first action of deconstruction of the ongoing configuration, thus the beginning of a microtransition. The analysis of the first two responses to the potentials for change allowed us to observe how families reconstruct stability. The analysis of microtransitions allowed us to explore how families deal with the change of interactions.

**Figure 2:**
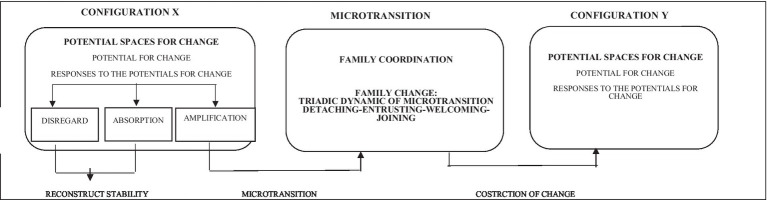
Coding system of TIAP.

TIAP provides a coding system which, through specific indicators of this interactive flow, allows to study the family functioning through the observation of the following processes: family stability, family change, family coordination, and the intra-family exploration (Figure 2).

*Family stability* is analyzed through the potentials for change of the family system (frequency and member enacting them) and through the types of responses implemented by the other family members that reconstruct the previous interactive configuration: disregard and absorption.

*Family change* is analyzed through the relational triadic dynamic of detaching-entrusting-welcoming-joining implied in the microtransitions that allows the deconstruction of a configuration and re-construction of a new one. Each of these processes is operationalized in term of verbal, corporal and expressive movements (Table 1). For each microtransition each process is coded in terms of occurrence/not occurrence and consistency/inconsistency of the communicative channels used (verbal, corporal, expressive). The presence of these 4 processes allows a family to build a relational context of safety that makes change possible (Cigala et al., 2018).

**Table 1:** The relational triadic dynamic of microtransition.

Processes	Definition
*DETACHMENT*	Verbal, corporal and expressive movements that allow one or more members to separate from the ongoing interaction and relate to other members, or choose the role of observer
*ENTRUSTING*	Verbal, corporal and expressive movements through which the active adult prepares the child for a new interactional involvement: the child can be left in one parent’s care (entrusted to one parent); in both parents’ care (jointly entrusted); or left to play alone (self-entrusted)
*WELCOMING*	Verbal, corporal, expressive movements through which a partner shows a willingness to become involved in the interaction
*JOINING*	Verbal, corporal, expressive movements through which the partners propose or consolidate a new interactive configuration

*Family Coordination* is assessed in each configuration and in each microtransition through the descriptors of attention, responsiveness, re-proposition of signals by all members (the signal of a member is rephrased and readdressed to the others) and contingency between responses. According to the combination of these descriptors, each family is ranked according to a four-point Likert type scale (present-very good/ present-good/discontinuous/absent) (Table 2).

**Table 2:** Levels of family coordination.

Very good	Good	Discontinuous	Absent
Attention and responsiveness by all members	Attention and responsiveness are completely present by two members or nearly completely by three members	Attention and responsiveness are present sometimes or they involve two members at a time	Absence of attention and responsiveness
Re-proposition by the system	Re-proposition by system is present when attention and responsiveness involve all members	Re-proposition by the system is absent	Re-proposition is absent
Contingency between responses is complementary	Contingency between responses is consecutive—fluid: some members start the process and the others follow it almost immediately	Contingency between responses is consecutive—difficult: the actions of the members take place in different times	Contingency between responses: rare—absent

The *intra-family exploration* is evaluated considering the number and the type of configurations that families can perform during the TIAP task. Through these indices it is possible to analyze the system’s ability to explore the various possible interactive scenarios in which a family member may be involved. The more scenarios family members can experience, the more exploration is allowed within the family. The number and type of explored configurations provides us with the information of the presence or absence of different family skills such as: the ability to explore the different forms of interaction within the family; the ability to interact with two people at the same time; the ability to stand on the periphery of the interactive scene; the ability to be in interaction with another, in the awareness of the presence of a third party.

Each of the indicators of the TIAP coding system (see TIAP coding grid; Table 3) allows three descriptions at three different levels: individual, systemic, and procedural. At the individual level we can describe the behavior of the individual, for example one parent is withdrawn from the interaction between the second parent and daughter. At a systemic level it is possible to observe how one’s behavior is complementary to the behaviors of the others. For example, the withdrawal of one parent corresponds to a particularly active behavior of the other parent and vice versa. At the procedural level it is possible to reconstruct the interactive dynamics that lead to a specific functioning at a given moment. In other words, it is possible to reconstruct how one’s behavior is the result of a relational dynamic that has developed over time. For example, we observe that parent1 enacts several potentials for change that both parent2 and daughter systematically ignore (disregard: fall into the void response); correspondently, we observe that parent1 stops attracting their attention and withdraws, while parent2 and daughter continue to play.

**Table 3:** TIAP coding grid.

Constructs	Indicator	Indexes	CONF I				MICROTR I				CONF II				MICRO II				CONF III				MICRO III				CONF IV			
			P1	P2	S1	S2	P1	P2	S1	S2	P1	P2	S1	S2	P1	P2	S1	S2	P1	P2	S1	S2	P1	P2	S1	S2	P1	P2	S1	S2
Family stability	Potential for change	Number																												
		To whom (P1/P2/S1/S2)																												
		Role (P_NP)																												
	Responses to the potentials for change	Type (ABS/AMPL/DIS)																												
		To whom (P1/P2/S17S2)																												
		Role (P_NP)																												
Family change	Triadic dynamic of microtransition	Detaching-entrusting-welcoming-joining (DET/ENT/WEL/JOI)																												
		Consistency/inconsistency C/I																												
Family coordination	Quality of coordination	Individual coordination Attention/responsiveness/contingency																												
		Family coordination Very good-good-discontinuous-absent																												
The intra-family exploration	Quality of exploration	Type of configuration (I/II/III/IV)																												

TO WHOM: Parent 1, Parent 2, Son 1, Son 2, etc.

ROLE: P, peripheral; NP, no peripheral.

TYPE: ABS, absorption; AMPL, amplification; DIS, disregard.

The execution of the task by the families is video recorded to allow an accurate and in-depth analysis of the material collected. The coding of the interactions is carried out by several observers who can be independent (experimental research context) or dependent (clinical and therapeutic context) (Kreppner, 2009).

TIAP has been used to evaluate parenthood and family functioning in cases involved in the legal context.

### 1.3 Parenting evaluation context

The concept of parenting can be defined as the generative capacity of an individual, understood in the Eriksonian perspective (Erikson, 1968) as the capacity to take care of someone other than oneself. As such, parenting does not coincide with biological generativity and has a relational and process-oriented nature. The parental functions derive from the relationships that the individual has experienced and experiences in everyday life and they develop over the course of the personal history and evolve constantly. Parenthood is not conceived as attributable to individual characteristics alone, rather as a function that emerges from the complexity of intra/extra-family relationships (Scabini and Cigoli, 2000; Bastianioni and Taurino, 2007). A further important aspect in the definition of parental functions is the concept of expanded parenting. It is based on the capability and possibility of parents to entrust their children to others who can play a positive role in the development of minors and to entrust themselves to others who can help them in the care of their children. Consciously accepting to take care of children in a broader context means enhancing parental functions by being able to rely on the resources available in this context (Venturelli, 2018; Fruggeri et al., 2023).

The Court’s most frequent requests regarding the evaluation of parenthood are: (1) assessing the personal characteristics of the parents and the child, (2) detecting the quality of the relationship between minor and parents, (3) identifying their parenting competence. In this perspective, the context of assessment of parenting is often connoted as an evaluation space clearly separate from that of intervention. However, in the court’s request for psychological assessment there is also a request, albeit sometimes implicit, to make a prognosis about the potential future functioning of the family system. Identifying and assessing a family’s specific resources, possibilities for change, and directions for change allows for a recoverability perspective. From this point of view, the space of parenting assessment can also become a space for containing and “transforming” parental conflict, and for activating the parents’ or family’s resources by promoting the achievement of shared solutions. According to Cigoli and Pappalardo (1997) the context of parenting assessment can represent a suspended time in which the family story can be better understood, and the resources can be activated to favor the restructuring of family relationships.

Conceiving the evaluation process as transformative implies some important reflections and methodological choices. Firstly, if the assessment should also give indications regarding recoverability, it becomes essential to provide in the evaluation process a space that includes the entire system involved. In this setting all the members can act the dynamics of interdependence and mutual coordination, and the psychologist or psychotherapist can interact with the whole family system, and evaluate the family overall resources, through the tools she/he considers most appropriate.

If the parenting assessment can also have a transformative value, the possibility of creating an alliance between the family and the clinician is fundamental. This alliance can be fostered if each of the member of family system involved can live the experience of “being together” and can perceive this context as a “transparent” and “fair” space, in which everyone is considered and informed, in order to reduce persecutory thoughts and fears of possible coalitions between subsystems (Escudero and Friedlander, 2017). To construct a transformative evaluation context of parenting, it is necessary to employ procedures that include some moments of “shared reflection” with the family, in which the clinician shares the meanings emerging during the sessions of family interaction. These meanings can help the family to read their relationships differently and to understand certain ways of functioning in a more circular and processual perspective.

## 2 Aim

The present paper focuses on the use of TIAP (*Triadic Interactional Analytical Procedure*) in parenting evaluation contexts and it aims to describe, through a clinical case, the main results emerged from a study conducted with families involved in a parenting assessment process.

In specific, the study is part of a broader collaborative project between the Child Psychiatry Unit (CPU) of the Italian National Health Service in Parma, the University of Parma and the Centro Bolognese di Terapia Familiare (CBTF). The main goal of this project is to highlight the potential of TIAP in the process of parenting assessment to identify specific aspects of family functioning that can give prognostic indications.

## 3 Participants

This project has currently involved 33 families involved in a parenting assessment process: 15 presented conflictual separation problems, while for the other 18 families episodes of violence were reported within the couple and/or toward the children. Twenty-seven families were seen at the CPU service and 6 were evaluated by the consultants of the CBTF. In general, the court involves the CPU professionals to evaluate parenting following reports of maltreatment of minors; while private consultants (like CBTF) are involved when one parent sues the other with the accusation of inadequacy in the management of the children.

All parental couples were heterosexual. Twenty-six families were Italian, 4 were foreign (1 South American, 1 African, 1 Eastern European, 1 Northern European), 3 mixed couples (2 Italian-African, 1 Italian-Moldavian). The ages of the 49 children ranged from 1 to 18 (all ages represented). 12 families had 1 child, 19 had 2 children, and 2 had 3 children. In 27 families the couple was divorced, of which 2 were remarried, 2 remarried than divorced; in 1 family with divorced couple, children were temporary in the custody of the paternal grandparents. Six families had cohabiting parental and marital couple, of which 1 was an adoptive family, 1 foster family, 1 family was composed of a single mother and foster parents.

The families participating in the study are characterized by a high variability both in terms of age of the children and cultural origin, as often happens in clinical research. Taking into consideration this variability, TIAP is a suitable method since both the procedure and the task can adapt to all cultures and all ages of children (Venturelli et al., 2022). Written informed consent to participate in this study was provided by the participants.

## 4 Procedure

TIAP was proposed to families together with other tools used routinely in parenting assessment protocols (Buone pratiche per la valutazione della genitorialità: raccomandazioni per gli psicologi, Ordine degli Psicologi dell’Emilia Romagna, 2009). The families involved were asked to sign the consent for video recording and research. The procedure was applied according to the described protocol. The recordings were analyzed by a team composed of psychological practitioners and researchers; all trained in the analysis of interactions according to TIAP.

To promote a context of trust and build a positive alliance, families were informed that the procedure was proposed to obtain the information necessary to help them overcome the current difficulties.

The team connected the results of the analysis with those emerging from the history of the family, thus providing the basis for the formulation of a hypothesis about family functioning. TIAP was applied for prognostic purposes: the team intended to give indications to the court highlighting both the dysfunctional aspects and the family’s resources to identify the necessary interventions to overcome the current critical issues. The analysis of family functioning resulting from the application of TIAP was shared with the families who were invited to reflect together on the inputs provided by the team. In this way, family members could begin to develop a reflexive ability, thus an awareness of their family functioning.

The research project was approved by all the centers (CPU of the Italian National Health Service in Parma, the University of Parma and the CBTF in Bologna) as well as by all the families involved.

## 5 Results

From the analysis of the 33 families, TIAP emerged as an extremely useful procedure in contexts of parenting skills assessment. In this article, one case will be presented in detail to exemplify the use of the method. The case was chosen because it allows to describe the various aspects of the family dynamic brought about by the application of TIAP that offer useful indications at a prognostic level, such as: the quality of co-parenting, the distinction between the marital and parental level, the role of the children. The description of the case includes how the information was returned to the family and the court.

### 5.1 George and Kate: marital couple or parental couple?

#### 5.1.1 Information from the family’s story

George and Kate met 19 years ago at the age of 50 and 30, respectively. She was single and he married, without children. She is a fashion designer, and he is an accountant, they first established a working relationship and then a sentimental one at the same time.

During their relationship, Kate’s desire for parenthood arose, George shared the same desire, as no children were born from the relationship with his wife. The years passed and the two became increasingly involved in the search for a pregnancy while at the same time George continued to be married and to live with his wife, with the promise to separate. Finally, when she is 36 and he is 56 the pregnancy arrives. A baby girl, Charlotte, is born. In the meantime, George spends a lot of time with Kate and the little girl, but he still lives with his wife. After a few months, another pregnancy unexpectedly arrives and after 13 months a boy is born: Aron.

A few months after the second birth, George reveals the situation to his wife, and she gives him her approval to take care of the children without divorcing.

Until the children are 5 years old, the situation proceeds in an ambiguous way: George spends a lot of time at home with Kate and the children, but he never moves in permanently and does not divorce his wife. Meanwhile, Kate puts him increasingly on the spot until, faced with the ultimatumt (either stay with us or leave), George decides to leave but to continue looking after the children. George returns to live permanently with his wife and asks for joint custody of the children, to which Kate objects; the judicial process thus begins. At first, the judge establishes a fifty-fifty joint custody, which Kate does not respect. According to her, the children do not want to go to their father because of inadequate relational attitudes on his behalf. Over the years, however, the children have always seen their father even at his home in the presence of his wife, but with many difficulties raised by Kate: obstacles to overnight stays, request for the presence of an external figure to protect the children. During this period, the children express a general unease about being with their father, particularly at his home.

Finally, George initiates new court proceedings to clarify the situation and to obtain effective 50/50 custody of the children. Nowadays, Charlotte is 13, Aron 12, George 69, and Kate 49.

#### 5.1.2 The court’s request

In front of the above scenario, the court makes this specific request:

“The court consultant shall listen to the children on the express delegation of the Court and update the situation of the family, verifying the developments that have taken place in the meantime, determining what is at present the most suitable placement and attendance of children in their best interest, and indicating the most appropriate modalities for the establishment of a meaningful relationship with both parents.”

The parties’ consultants appointed by George’s and Kate’s lawyers agree on using TIAP as suggested by the Court’s consultant to have a better understanding of the relational dynamic among the four of them, a closer look at the relationship of children with each parent, and a more specific analysis of the relationship of George and Kate both as co-parents and as ex-partners.

#### 5.1.3 The information about the family before TIAP

The psychologist and the social worker of the child protection service, the Court’s and the parties’ consultants are the professionals involved in the case. The following is the information they collected during several conversations with each parent and each child and passed on to the professionals in charge of TIAP.

Kate is convinced that she must defend her children from George who is considered by her totally inadequate. She constantly blames her ex-partner and points out his shortcomings.

George claims that his difficulties in playing the parental role depends on Kate’s lack of legitimization, and on the impediments that she imposes on him in the everyday management of their children.

The psychologist saw Aron as a very inhibited and insecure young boy who appears somewhat stuck at a developmental stage prior to his age, both physically, emotionally, and cognitively. He speaks in a very low tone of voice, his answers are evasive, sometimes he even refuses to respond to the professionals’ questions. According to the psychologist’s observations, the mother adopts a symbiotic mode with Aron; she exclusively points out his frailties and problematic aspects, thus hindering his process of identification. Aron speaks of his father as someone who is not very playful, who sometimes teases him; Aron feels little considered by his father, but he does not report any detrimental experience. The results of the Double Moon test (Greco, 1999), show that Aron does not include his father in the circle of the significant persons. For these reasons, the social worker suggests helping the father to adopt more appropriate behaviors and attitudes toward his son to connect with him at a deeper interpersonal level. The social worker thinks that the negative judgment that Aron expresses toward his father comes from experiencing a constant conflict between his parents, and that it could change if he could have a different relationship with him.

Charlotte appears to the psychologist and to the social worker very contradictory in describing both her own and her brother’s feelings when spending time with their father: some descriptions are positive, and some others are negative. However, when the professionals ask for details, no distressful episodes are referred.

The psychologist, who has been following Charlotte for about a year, reports that Charlotte has always attended the meetings but has maintained a rather superficial level in the discussion of topics concerning the intimate and family sphere: she immediately made it clear that she did not want to talk about her relationship with her father. From the conversations with Charlotte, the psychologist understands that the girl appreciates the current family organization, in which the children see their father little and do not stay overnight with him. Charlotte struggles to delve into the personal and intimate area of her life, focusing rather on topics pertaining the extra-familiar area. According to the psychologist, Charlotte also tends to take a complacent attitude toward professionals and her father, whom she generally tries to please by showing a smiling and cheerful attitude even in uncomfortable situations.

According to the psychologist’s report, the children are involved in the parental conflict in which they feel compelled to take a side.

No information is reported by the professionals about how children see their mother.

#### 5.1.4 The analysis of family interactions from TIAP

Family members arrive on time and together. They willingly agree to play and engage in the proposed Lego activity. The family sits spontaneously around the table according to this arrangement: father, Charlotte, Aron, mother. Before the end of the handover, the mother asks for some specifications on the various steps of the procedure. Father and mother negotiate who starts playing first, without involving the children. At first, both invite each other to start, then the mother in a sarcastic tone urges the father and he takes an active role.

Family stability: In the first configuration (11 min), father is active in playing with the children while the mother takes a peripheral observer position. The children are composed and silent, sitting very close together, they carry out their own constructions (each their own) with some reciprocal exchanges. Charlotte responds to her father both verbally and with brief exchanges of glances, albeit with little dialog. Aron ignores his father, does not respond to him, sometimes makes barely uttered sounds; at the same time, he constantly looks at mother and she returns the glances. During this configuration, an interactive dynamic emerges in which the father, not receiving responses from Aron, addresses him in an increasingly insistent manner; when he does this, mother intervenes and enters the game, Aron stops playing, the father responds to the mother and the children resume playing together thanks to Charlotte who invites her brother back to play. In this configuration, the potentials for change are: Aron’s toward his mother (many shared glances while the father is talking) and they are absorbed; the mother’s abandoning the peripheral position to enter the game when the father becomes more direct and pressing toward Aron; mother toward father reminding him that soon it will be her turn to play (“when you are too desperate you tell me that I will play”); the answers are absorbed by Aron e and sometimes Geroge and ignore by George. Charlotte never makes any potential for change and never looks at her mother. In general, the emotional climate is poor, there are no moments of sharing and understanding between the active participants in the interaction.

In the second configuration (6 min), the mother plays with the children, and the father is in a peripheral position. The emotional climate changes as soon as the mother starts playing with the children; Aron becomes more active, he moves and plays in a more engaging way, he has several verbal exchanges with the mother who addresses both children together and individually. In this configuration the potentials for change are made by father, who maintains a peripheral position with great difficulty: he intervenes often, moves around, plays alone. In the rare moments when he observes, he has a sad and withdrawn expression. The only one who pays attention to him and absorbs his potentials for change is sometimes Charlotte, while the others always ignore him.

In the third configuration (5 min), they are supposed to play together, which is not what happens, as they are never all active, connected and interacting. Sometimes the mother is in interaction with Aron while Charlotte is interacting with the father, thus creating two parallel dyads; sometimes the children play with the mother and the father plays alone; finally, there are brief moments in which mother and father talk and the children play.

In the fourth configuration (7 min), the task requires that children play together while the parents talk. The atmosphere is relaxed, and the siblings play together with involvement. The mother turns to the father, looking at him and starting a conversation, the father responds but shifts his gaze to the children and makes numerous potentials for change by inviting them into the conversation. The potentials are ignored by Aron who continues to play quietly and are occasionally absorbed by Charlotte; more often it is the mother who brings the father back into the conversation with her. In general, the emotional climate is positive and there are shared looks and positive emotional attitudes especially from the mother toward the father.

Family change. The first microtransition (from father playing with children to mother playing with children): After 5 min of the father playing with the children, the mother makes a first potential of change by saying “when you are too desperate, you tell me and I’ll play,” to which the father does not follow up, and the ongoing configuration continues for another 5 min; then the father says to the children “eventually, you could continue this with mom” while continuing playing; the mother responds “I was beginning to despair, the best Lego pieces are all gone” but she does not enter the game. Both parents show an incoherence between verbal and nonverbal behaviors. Six minutes from the previous potential, the mother says, “who’s going to let me play?” ironically looking at the father who replies, “I’ll let you play,” but he continues to move the Lego pieces and talk. At this point the mother looks at the Lego box and says to the father “can you bring it a bit closer to me?” the father replies “sure” and begins to explain what they have been doing so far, thus remaining active. In the meantime, the children stay still. The father continues to address the children, the mother in turn begins to interact with them. Both children respond to her promptly and an interaction starts between the three of them (the children’s welcoming of their mother and a mutual involvement is observed) while the father continues to intervene (father’s difficulty in detaching).

The second microtransition (from mother playing with children to playing all together) is preceded by a preannouncement from the mother who after 5 min of playing with the children says “afterwards we have to play together with daddy” and turning to the father she says ironically “you do not want to do anything, do you?, just watch us work …” the father replies “I worked before …,” meanwhile he takes some pieces from the table and passes them to Charlotte. At that point, the father proposes to use some pieces for a new construction while the mother is talking to Aron, Charlotte responds to the father facilitating his involvement (Charlotte welcomes the father).

Finally, in the last microtransition (from playing all together to children playing alone and parents talking) there is another preannouncement from the mother who after about 5 min says to the children “in a little while you guys play alone so we can rest” and continues to play, the children do not say anything and the father says to the children “when you say you are ready we take off”; the mother laughs, Charlotte looks amazed and the father says “Aron are you ready to be an architect?” Aron does not answer and the father asks, “Yes, or no?” Aron with a thread of voice says “no” and the father says, “but we are here.” They all continue to play. Meanwhile the mother talks to the father about a film, calling him by his nickname (the mother welcomes the father) and the father continues to talk to the children. Then the father says, “now we’ll let them play the final part” and stops playing (father’s detachment), in the meantime the mother has also stopped playing (mother’s detachment) and continues to address the father by looking at him, the father speaks loudly, gesticulating and keeping the children inside the conversation.

All moments of transition are difficult for this family and are led by the adults; the children are never involved nor verbally guided by their parents. The mother takes the lead in proposing the change of configuration (she also decided who had to start playing from the first configuration). A high degree of incoherence is observed between the verbal and nonverbal language on the part of both parents, who, for example, verbally propose a change in the configuration remaining in the same position.

The moments of microtransition for this family are characterized by the absence of the construction of a safe context, as the entrusting process is rarely present and mainly incoherent. Welcoming is only present from the children toward the mother; from Charlotte toward the father (never from Aron); from the mother toward the father but only in the configuration in which they talk to each other and not when they all must play together.

Family Coordination is discontinuous. During microtransitions, attention and responsiveness are not shown by all members and are only present at times. Neither one of the parents re-proposes the other parent’s signals to the children. Contingency between responses is consecutive– difficult: the actions of the members take place in different times. A total lack of attention and responsiveness by Aron toward his father is observed during the configurations. Both parents are attentive toward their children but the lack of reciprocity between father and son and the lack of re-proposition by the other parent makes exchanges difficult in both the first and third configurations.

Intra-family exploration: The family explores all configurations but with different specificities. In the first configuration Aron does not play with his father and there are different potentials for change toward and from the mother. Charlotte responds to the father’s urging and has a facilitating role in bringing Aron back into playing. In the second configuration mother and children play together and the father struggles to remain in the peripheral role of observer. In the third configuration there is never a four-way game but either two parallel dyads (father and Charlotte; mother and Aron) or mother and children with the father playing alone. In the fourth configuration the children play with each other, and mother and father talk with numerous potentials for change enacted by the father who tries to call the children in.

Comment: At the end of the game, the psychologist asks how they felt during the procedure, and they all answered that it had been a positive experience.

#### 5.1.5 Reflection and hypothesizing starting from the observational data

The following aspects emerge from the administration of TIAP. The analysis of the interactions reveals certain relational dynamics and redundant roles.

Aron’s rejection of his father: Throughout the game, Aron does not interact with father, he timidly answers when faced with the strong pressing from him. On the contrary, he talks and plays with both mother and sister. What is Aron saying with this behavior? Moreover, Aron looks at the mother every time the father seeks for a contact with him, and when the father becomes more directive, the mother intervenes interrupting the interaction between the two. On the other side, the mother never facilitates and supports the interaction between the father and the children.

Charlotte’s appropriateness: In all configurations Charlotte is the only family member who has a role appropriate to the task. She accepts to have an interaction with her father, with her mother, with her brother, and with both parents together, but she is focused on the activity, without any exchange or sharing emotional connection with them.

It seems that the two siblings have taken dichotomous roles: Aron obstructs the relationship with their father while Charlotte fosters it, one divides the family and the other connects it while the parents do not build a safe context for these children within which to explore various relationships. Aron in his role is at the center of both mother’s and father’s attention, Charlotte is less central.

Inconsistency of messages from parents to children is detected at different times during all configurations and particularly during microtransitions. These are messages that simultaneously convey different content: at the verbal level, one parent expresses an intention to engage the other parent, but at the nonverbal level does not change his or her position to allow the other to enter, who in turn does not follow up on the proposal. This dynamic facilitates neither family coordination nor the construction of a safe context for children.

Kate’s role as a mother and as a partner: analysis of the interactions reveals a different way Kate relates to George when they are in the parental position and thus in interaction with the children or when, as in the last configuration, they are prompted to relate in the absence of the children. Kate appears very likely to accommodate George when the children are playing alone and in parallel seems to disregard him when he is in the parental role.

George is ignored as a father by both Aron and Kate, and in parallel he tends to become pushy by enacting interactive modes that are not always appropriate. Both aspects can become the cause of each other fueling a negative vicious cycle.

Comments:

To increase the well-being of the children and of the system in general, we think it is useful to work with these parents on the separation of the marital and parental axes. How does the story of their relationship affect their parenting today? Mother seems at ease to interact with George alone (as in the fourth configuration), but not when he takes on his paternal role (as in the first and third configurations). On the other side, the father seems to be focused on his involvement with the children yet showing no interest in a direct interaction with Kate (see last configuration). Where do George and Kate stand with respect to processing their separation? Is it possible that the conflict between them comes from the fact that while George is concentrated on the relationship with his children, she is interested in clarifying her relationship with him as former partners? It could be useful to work on these issues with the two parents to distinguish between the two levels: that of parenting where a coordination and recognition of each other’s roles are fundamental, and that of the marital relationship which is defined as finished but probably not yet completely processed from an emotional point of view.

Another aspect, which we consider important and complementary, concerns the building of the paternal role. This implies working with mother to remove the possible psychological conflict that prevents her recognition of George’s role. Working with George to become more sensitive to his children’s emotional needs.

Individual support for Aron and Charlotte to help them to cope with the situation while their parents work to restore a parental collaboration.

The analysis of Tiap was first shared with the professionals involved in terms of an overall analysis of the situation showing the possible future positive evolution for all. Then the Court’s consultant sent his report to the judge.

#### 5.1.6 Return to the court

This is a synthesis of the analysis that the Court’s consultant reported to the judge.

The two parents are in serious relational difficulty toward each other, both heavily involved in their personal conflict. Neither parent is currently able to disentangle him/herself from the conflict, so it would be useful and necessary to help them to process their difficult history, right from the start.

The parents never managed to communicate together to the children their decision to separate, nor they ever reassured them about the continuation of parenting on behalf of both. This has certainly affected the children’s experience from an early age. A restoring of co-parenting is essential for the children; thus, parents must see experts that help them to reach this goal. A greater presence of George in children’s life (as he desires) could be positive for both the children and Kate, but this is possible only after a restoration of their relationship as ex-partners.

The goal “to establish a meaningful relationship of children with both parents” makes it necessary to consider and intervene on the following aspects:

Children do not perceive the mother’s trust on father. This does not build a secure relational context, which would instead facilitate the creation of a reassuring and evolutionary relational environment for the two children and for the separated family itself.

George and Kate do not seem aware of the importance to invest in their relationship as parents. Reflection and attention on this issue should be shared with both.

There is a lack of facilitation and support for interaction between the father and the children on Kate’s part, without which the children do not feel legitimized in their relationship with their father.

A key element contributing to the breakdown of the family’s relational dynamic is the lack of coordination between the two parents. This lack prevents the construction of a bridge from each parent to the other, highlighting an absence of collaboration, and preventing the transmission to the children of the concept of co-parenting, which, in fact, is currently absent.

Given the current emotional state of the children, they should continue to stay with mother, with the possibility of father to spend time with them that should gradually increase according to some interventions that include:

Psychological support for both children, who live a precarious and difficult emotional condition.

Massive support and constant monitoring from the Social Services with the aim of working directly and indirectly on the above-mentioned relational aspects.

A parental coordinator acting in support of the Social Services’ work about the above mentioned psychological and relational issues.

Without these interventions the psychological conditions of the children may further deteriorate and until their accomplishment it is not possible to envisage a definitive custodial agreement for which a new further assessment may be necessary in the future.

## 6 Discussion

As evidenced by the analysis of the case, an added value of TIAP over other instruments used in the assessment of parenting skills is that it offers the possibility of simultaneously observing the family at different levels: the individual, the subsystems, and the whole system. These different levels represent complementary points of view that allow the clinician to reconstruct a systemic and complex understanding of family functioning, escaping the unidirectional and linear causal logic that induces the search for the “culprit” that families under evaluation often propose and with which professionals risk colluding.

In this sense, the possibility to analyze co-parenting is certainly among the strengths of TIAP. In evaluating parents’ skills, co-parenting is often not directly observed but “reconstructed” from the cross-referencing of the results coming from individual instruments administered to each parent. Instead, TIAP allows to observe how the parents coordinate while they interact, thus allowing to describe the process, the circularity and the interdependence involved in such an interactive situation. In addition, the procedure, as shown very well in the case analyzed, makes it possible to analyze the role of each parent in facilitating or not facilitating the children’s access to the other parent (see for example the different degree of coordination between parents in each specific configuration and the presence or absence of the entrusting process).

As noted by Margolin et al. (1998), relying on narratives and accounts, individual self-report methods collect retrospective and global (general) descriptions of the phenomenon under study; observational methods, instead, allow the moment-by-moment description of the process as it takes place. In the case described above, for example, the analysis conducted thanks to TIAP allowed to detect relational aspects that had not emerged from the individual interview conducted with the family members before the administration of TIAP. During the interviews, Kate described a conflictual relationship with George who emerged as an untrustful and negative parent. During TIAP it was possible to observe the positive relational attitude of Kate toward George when the procedure asked them to interact independently from the children. This is what led the professionals think that if they wanted to work with them as parents, they had first to make sure that the couple processed the end of their relationship as a marital couple to avoid the dysfunctional dynamic of “negotiating children for the couple relationship.”

Moreover, as reported by the psychologist and the social worker, both children appeared particularly reticent to express themselves regarding family issues during the individual interviews. Thanks to the administration of TIAP, the importance and specificity of their role within the family dynamic emerged very clearly. In particular, the professionals that had interviewed the children before TIAP had noted that the children did not want to meet their father, without though identifying any specific distressful episode or particularly harmful attitude on father’s side, thus concluding that an intervention was needed to help him to learn how to deal with his children’s emotional status. During the application of TIAP, though, it became clear how Aron’s rejection of his father was part of a larger family dynamic: the father urges Aron to play together; Aron refuses the father’s invitations to interact; the father awkwardly insists to involve him; the mother intervenes and interrupts the interaction. Charlotte invites his father to play. This is a dynamic that could never be reported by the family members since they participate in it without being aware. Through TIAP the “voices” of children become clear within the context of family relationship: what cannot be said, it is shown (Anolli, 2002).

The analysis of the triadic relational dynamics of detaching/entrusting/welcoming/joining allowed to evaluate the resources of the system with respect to the possibility of co-constructing a safe family relational context which enables parents and children to deal with separations in a secure way (Byng-Hall, 1995b). This dynamic recurs in daily experience when children pass from the custody of one parent to the another or to other caregivers. These moments often turn out to be critical events for families with divorced parents. In the assessment of parental competence, the process of entrustment of children by one parent to the other is an important indicator of the capability/incapability of parents to cooperate to help the child to cope with change.

In the TIAP approach, family stability and change are not conceived as opposite poles of a continuum, but as different processes that can be analyzed through different indicators. In this sense, TIAP makes it possible to separately evaluate the abilities of family members to stay in certain configurations and those to change, thus allowing a specific analysis of criticalities and resources. For example, our study highlighted how through the TIAP index of intra-family exploration, it is possible to identify which family interactive spaces can be practiced by family members and which not. This information has proved to be particularly useful in orienting the judge’s decision regarding the type of parental custody, on one side; and to indicate the area of relationships that should be supported with a psychotherapy, on the other.

From a general overview of all the cases analyzed emerged that TIAP has proven to be an inexpensive method of observation, implying a shared, involving task, perceived as low-stressful and low-judgmental by both parents and children, who can perceive themselves as active and competent participants (Venturelli et al., 2022). The analysis of all cases through TIAP highlighted some strengths of the method, which in particular allowed:

1. To *focus on parental resources*, where clinical interviews and self-report tests have mainly identified criticalities; this allowed the psychologist in charge of the case to give a prognostic opinion to the family and to the Court.
2. To *involve in the procedure significant figures others than parents*, enabling to conduct trigenerational or multinuclear families analyses and to understand parents’ difficulties in such a context, but also to identify eventual resources for future interventions. For example, in four cases it was possible and necessary to involve in the procedure other significant persons present in the children’s lives: the father’s partner; the grandmother; the foster parents together with the biological parents. In particular, in foster care situations, TIAP can be very useful for analyzing the complex relational triadic dynamic involving the foster child (cf. Greco and Iafrate, 2001). Specifically, in two cases, TIAP was applied to a foster situation of two children aged 2 and 5 involving the biological mother and the foster parents, through two observational moments: a first triad formed by biological mother, children, foster mother and a second triad formed by biological mother, children, foster father. The involvement of both biological and foster parents in the procedure allowed for the observation of the dynamics of mutual trust between the different family units, and the children’s role in this internuclear dynamic. In applying TIAP, it is possible to expand or narrow the observed system, creating a different focus and connecting parts that are in danger of not being seen or of remaining isolated from each other.
3. To *analyze the quality of co-parenting* with respect to the following specific processes: family coordination, the ability to foster and build a safe context, the accessibility to the other parent and the possibility to explore certain family interactive configurations.
4. To *give voice to children*, whose subjectivity is recognized on a par with everyone else’s, highlighting their active role within family dynamics. This both in cases where children were very young and had difficulty expressing themselves verbally, and in situations where children or teenagers had already expressed their views through interviews or other tests, allowing through the observation of family relationships to substantiate that information and give it relational meaning.
5. To *collect information that can be used by the professionals* of the child protection tea*m* (in the public sector) or by the Court’s and parties’ consultants (in the private sector). These professionals relying on the analysis of the whole family, can avoid the iatrogenic position of colluding with the conflictual dimension of the family by taking sides with one or the other part involved.

In all cases, the evaluation of the parenting skills conducted through TIAP has shown how this procedure constitutes both an evaluation tool and a tool for building a therapeutic alliance (Escudero and Friedlander, 2017). The administration of TIAP allows the clinician to observe family relationships in action, and the family members to observe themselves. The “play” represents, in fact, an “acted out and co-constructed plot” accessible to all members of the family system, based on which the clinician builds her/his narrative of the family also integrating what emerged from the administration of other individual tools. This narrative or clinical hypothesis, being able to rely on a plot shared by all family members, allows them to feel seen and to recognize themselves in a shared narrative.

The results of the application of TIAP can be shared within a network of professionals with different functions and roles (Fruggeri et al., 2023, cap.9). The collected data shed light contemporary on the individuals, the dyads, the whole family and on how these different levels of the system intertwine, thus returning to the network of professionals (court-appointed technical consultant, party technical consultants, social workers, educators, psychologists, etc.) a complex picture which offers the context for understanding in a non-blameful way the functioning of the system, avoiding possible collusion of the group of consultants with the family conflicts. TIAP does not deny the information collected from different instruments, it allows to understand them within a larger context.

Given these reflections derived from the case analyses, we believe that it would be desirable to continue the research by increasing the group of participating families and by involving a greater number of families belonging to different cultural contexts, as well as including family systems with same-sex parents. A systematic analysis of the functioning of these families would allow us to verify whether the TIAP procedure, could be reliable, in terms of both the proposed task and coding system, to evaluate parental functioning in different family forms. This direction of research could be very interesting in the clinical field, because it would allow to verify whether TIAP can overcome the limitations of many clinical tools used in the evaluation of parenting skills which are strongly influenced for example by cultural variables or gender stereotypes (Gato et al., 2013).

## Data availability statement

The raw data supporting the conclusions of this article will be made available by the authors, without undue reservation.

## Ethics statement

The studies involving human participants were reviewed and approved by the Child and Adolescent Neuropsychiatry Clinic of the Italian National Health Service in Parma and the Bologna Family Therapy Center. Written informed consent for participation in this study was provided by each participant, in the case of minors present in the family, written informed consent of their parents or legal guardians/next of kin was obtained. Written informed consent was obtained from the individuals or legal guardians/next of kin for the publication of any potentially identifiable data included in this article.

## Author contributions

AC: Conceptualization, Data curation, Investigation, Methodology, Supervision, Writing – original draft, Writing – review & editing. EV: Conceptualization, Data curation, Investigation, Methodology, Writing – original draft, Writing – review & editing. LF: Conceptualization, Data curation, Investigation, Methodology, Supervision, Writing – original draft, Writing – review & editing.
